# A Text Book of Hygiene

**Published:** 1885-02

**Authors:** 


A TEXT-BOOK ON HYGIENE. A comprehensive treatise on the principles and practice of preventive medicine from an American stand-point, by George H. Rohe, M. D., Prof. of Hygiene, College of Physicians and Surgeons, Baltimore; member of the American Public Health Association; of the American Dermatological Association; of the medical and chirurgical faculty of Maryland; Corresponding member of the New Orleans Academy of Sciences, etc. Baltimore: Thomas & Evans, 1885.

This common sense view of most points involved in the preservation of health is rid of much cumbrous bibliographical history, which usually besets the reader of latter-day productions; and we accept instead, with satisfaction, the foot notes referring to authorities upon definite subjects.

Those who desire practical information in regard to the different phases of hygiene touching the individual, the family, the school, society, hospitals, camps, prisons, etc., will find the data of most utility concisely presented in this work, and while the author does not indulge in far-fetched hypotheses he gives a reason for his faith by a plain and clear exposition of the principles involved in the various hygienic measures recommended for the adoption of mankind. While there are some very trite and common-place remarks made in connection with the ordinary affairs of life which may strike the reader as axiomatic truths, yet it should be remembered that a book which is intended for all students, practitioners and sanitary officers, must contain elementary as well as more recondite data—so as to cover the entire field of exploration.

There is not, perhaps, in the English language, any other production of 300 pages having so much valuable matter connected with the prevention of disease, as is contained in this text book of hygiene, by Dr. Rohe of Baltimore.

